# Bioinformatics Analysis Reveals Biomarkers With Cancer Stem Cell Characteristics in Lung Squamous Cell Carcinoma

**DOI:** 10.3389/fgene.2020.00427

**Published:** 2020-05-13

**Authors:** Yi Liao, Hua Xiao, Mengqing Cheng, Xianming Fan

**Affiliations:** Department of Respiratory and Critical Care Medicine II, The Affiliated Hospital of Southwest Medical University, Luzhou, China

**Keywords:** lung squamous cell carcinoma, cancer cell stemness, prognosis, WGCNA, TCGA

## Abstract

**Background:**

Tumor stem cells play important roles in the survival, proliferation, metastasis and recurrence of tumors. We aimed to identify new prognostic biomarkers for lung squamous cell carcinoma (LUSC) based on the cancer stem cell theory.

**Methods:**

RNA-seq data and relevant clinical information were downloaded from The Cancer Genome Atlas (TCGA) database. Weighted gene coexpression network analysis (WGCNA) was applied to identify significant modules and hub genes, and prognostic signatures were constructed with the prognostic hub genes.

**Results:**

LUSC patients in the TCGA database have higher mRNA expression-based stemness index (mRNAsi) in tumor tissue than in adjacent normal tissue. In addition, some clinical features and outcomes were highly correlated with the mRNAsi. WGCNA revealed that the pink and yellow modules were the most significant modules related to the mRNAsi; the top 10 hub genes in the pink module were enriched mostly in epidermal development, the secretory granule membrane, receptor regulator activity and the cytokine-cytokine receptor interaction. The protein–protein interaction (PPI) network revealed that the top 10 hub genes were significantly correlated with each other at the transcriptional level. In addition, the top 10 hub genes were all highly expressed in LUSC, and some were differentially expressed in different TNM stages. Regarding the survival analysis, the nomogram of a prognostic signature with three hub genes showed high predictive value.

**Conclusion:**

mRNAsi-related hub genes could be a potential biomarker of LUSC.

## Introduction

Lung cancer ranks first in morbidity (11.6%) and mortality (18.4%) according to the latest worldwide survey of 20 regions from five continents and is the leading male cancer in both developed and developing countries (Bray et al., 2018) among all cancers. In February 2018, the latest national cancer statistics released by China’s National Cancer Center revealed the same grim situation: lung cancer is still the most common malignant tumor in China in terms of morbidity and mortality. Lung cancer is also the leading cause of death from malignant tumors in all regions of China (Chen et al., 2018). According to different histopathological characteristics, lung cancer is divided into non-small cell lung cancer (NSCLC) and small cell lung cancer (SCLC), and lung squamous cell carcinoma (LUSC) is one of most common subtypes of NSCLC (Barlesi et al., 2016).

Research on the pathogenesis and pathological mechanism of NSCLC is still controversial; some studies (Friedmann-Morvinski and Verma, 2014; Leon et al., 2016; Shibue and Weinberg, 2017) have shown that tumor stem cells play important roles in tumor survival, proliferation, metastasis and recurrence. For example, a recent study used bioinformatics methods found that *FOXM1* and *MYBL2*, which are involved in the process of cell proliferation, can be used as potential biomarkers and therapeutic targets of NSCLC (Ahmed, 2019). This theory provides a new direction and idea for us to understand the origin and nature of the tumor and clinical treatment. In essence, tumor stem cells maintain the viability of tumor cell populations through self-renewal and infinite proliferation. The movement and migration capacities of tumor stem cells make the metastasis of tumor cells possible. Tumor stem cells can remain dormant for a long time and express a variety of resistant molecules but are not sensitive to the external physical and chemical factors that kill tumor cells. Therefore, tumor stem cells provide a new direction and visual perspective for us to re-understand the origin and nature of tumors, as well as clinical tumor therapy.

In addition, based on the theory of tumor stem cells, some scholars have introduced a new concept — stemness indices (Malta et al., 2018). The expression profile and methylation data of different tumor samples were collected from The Cancer Genome Atlas (TCGA) and other public databases. An innovative one-class logistic regression machine learning algorithm (OCLR) (Sokolov et al., 2016) was used to extract transcriptomic and epigenetic feature sets derived from non-transformed pluripotent stem cells and their differentiated progeny. Two independent stemness indices, the mRNA expression-based stemness index (mRNAsi) and the epigenetically regulated-mRNAsi (EREG-mRNAsi), were calculated. Among them, the index range was 0–1; the closer the value was to 1, the stronger the stem cell characteristics of tumor cells.

With the continuous development of high-throughput sequencing technology, it is convenient to explore the occurrence and development of tumors at the genetic level and to identify possible therapeutic targets (Goldfeder et al., 2017). Traditional methods use differential expression detection to identify potential biomarkers but may miss useful genes. Therefore, weighted gene coexpression network analysis (WGCNA) (Langfelder and Horvath, 2008) was adopted in the current study. The WGCNA was based on two hypotheses: (1) genes with similar expression patterns may share common regulatory networks and/or functional correlations or be involved in the same pathway; and (2) the gene network conforms to scale-free distribution. Based on these two hypotheses, the gene network can be divided into different modules according to expression similarity, and hub genes can be identified.

The purpose of this study was to obtain modules that are closely related to stem cell characteristics and to further identify the hub genes located in the regulatory center with the help of high-throughput sequencing data from a public database and the WGCNA method. We also determined whether these genes have a clear effect on prognosis.

## Materials and Methods

### Data Processing

The flow diagram of our study was shown in Supplementary Figure S1. Level 3 RNA-seq data (HTSEQ-FPKM-UQ) and clinical information were downloaded from the TCGA website^1^. Ensembl IDs were converted to gene names via the Ensembl database^2^, and log2 processing of the data was performed. If a gene had multiple expression values, they were averaged. Each sample from the TCGA and its corresponding mRNAsi and EREG-mRNAsi data were downloaded from https://www.ncbi.nlm.nih.gov/pmc/articles/PMC5902191/. Any samples that were missing the stemness index or clinical information were excluded.

### Correlation of the mRNAsi and Clinical Characteristics

Differences in the mRNAsi between normal and LUSC tissues were compared using the unpaired *t*-test. Relapsed and non-relapsed patients who did not receive adjuvant therapy were also compared based on their mRNAsi. One-way ANOVA was used to compare significant differences in the mRNAsi between groups of variables. GraphPad Prism version 7 (64 bit) was used to perform the above analysis. To compare differences in prognosis, two indicators were evaluated: overall survival (OS) and progression-free survival (PFS). OS was defined as the time between the diagnosis of a tumor and death from any cause. PFS was defined as the time between the diagnosis of a tumor and the time to progression (in any form) or death from any cause. X-tile software (version 3.6.1) (Camp et al., 2004) was used to determine the best cut-off value in the survival data. The working principle of this software is to group different values as truncation values for the statistical test, and the result with the smallest p value is considered the best truncation value. Kaplan–Meier analysis was performed, and the *p*-value for two groups was calculated by the log-rank test with the survminer package in R software (v 3.6.0). *P* < 0.05 was considered a significant difference.

### Differentially Expressed Genes (DEGs)

The limma package (Ritchie et al., 2015) was used to identify DEGs between LUSC and normal tissues. The inclusion criteria for DEGs were log2-fold change (FC) > 1 and adjusted *P* < 0.05.

### Weighted Gene Coexpression Network Analysis

#### Construction of a Coexpression Network

We used the DEGs obtained in the previous step to construct coexpression networks with the WGCNA package (Langfelder and Horvath, 2008) in R software (v3.6.0). The *goodSamplesGenes* function was used to determine whether the sample data were complete. It was also used to perform sample clustering to identify and remove outliers. Pearson correlation coefficients between each group of genes were also calculated, and their absolute values were used to construct the gene expression similarity matrix according to the following formula: a_*ij*_ = |cor (x_*i*_, x_*j*_)|^β^, where x_*i*_ and x_*j*_ represent nodes i and j of the network, respectively. A β value was selected to build the proximity matrix so that gene distribution conformed to a scale-free network based on connectivity. The adjacency matrix and topological overlap matrix (TOM) were constructed after obtaining the β value. The TOM obtained was then clustered by dissimilarity between genes, and the trees were then cut into different modules by the dynamic shear method (the minimum number of genes in the module was 50). Some modules were combined according to the correlation coefficient.

#### Identification of Significant Modules

We selected the hierarchical clustering module that was the most closely related to the mRNAsi and EREG-mRNAsi for further analysis. Genetic significance (GS), module significance (MS) and the module eigengene (ME) were also calculated. GS was defined as the level of correlation between gene expression and the mRNAsi and EREG-mRNAsi. The calculation method used was the log10 transformation of the p value in linear regression. It represents the relevance of each gene in the module to characteristics. MS was defined as the average of significance of all genes in the module. We merged similar modules using a cut-off value 0.55, and then the modules that had the largest MS were considered the most sample trait-related modules. ME was defined as the first principal component obtained by principal component analysis of the gene expression matrix of each module. Among all the modules, the module with the highest MS was considered to be related to the mRNAsi and EREG-mRNAsi and was selected for further research.

#### Identification of Hub Genes

The GS and module membership (MM, correlation between the module’s own genes and gene expression profiles) of each gene were calculated after defining significant modules. The stronger the correlation was between the genes and significant modules, the stronger their relation to the stemness indices. Therefore, the inclusion criteria for a hub gene were set as follows: MM > 0.6 and cor. gene GS > 0.4.

#### Functional Enrichment Analysis

The clusterProfiler package (Yu et al., 2012) was used to perform functional enrichment for Gene Ontology (GO) and Kyoto Encyclopedia of Genes and Genomes (KEGG) analyses of the selected module. GO analysis consists of three terms: biological process (BP), cellular component (CC), and molecular function (MF). An adjusted *P* < 0.05 was used as the threshold.

#### Relationships and Interactions Among Hub Genes

STRING (version 11.0)^3^ is an online database that can be used to study and visualize the network of interactions among proteins. Coexpression relationships among the hub genes were calculated based on gene expression levels to determine their strength at the transcriptional level. The Pearson correlation between genes was calculated using the R corrplot package^4^, and the correlation matrix was visualized.

#### Validation of Hub Genes

GEPIA^5^ is an online site that allows differential expression profiling, pathological staging, and patient survival analysis of tumors and normal tissues in the TCGA database. GEPIA data are derived not only from the TCGA database but also from the sequencing data of normal tissues in the Genotype-Tissue Expression (GTEx) project^6^ (Tang et al., 2017), which compensates for the shortage of normal tissue samples in the TCGA database. Therefore, this database was used to verify whether the expression of the hub gene was higher in tumor tissue than in normal tissue and whether the hub gene was differentially expressed in different TNM stages.

### Survival Analysis

#### Establishment of the Prognostic Signature

The relationship between each hub gene’s expression level and OS was assessed by univariate Cox regression analysis, and hub genes with *P* < 0.05 were entered into the multivariate Cox regression process using the Akaike information criterion (AIC) (Yamaoka et al., 1978). A risk score formula was created using the corresponding data obtained through multivariate Cox proportional hazards regression analyses with the hub genes whose *p*-value was <0.05. In Equation 1, n denotes the number of prognostic hub genes, Gi represents the expression value of the ith hub genes, and weight i denotes the coefficient of each significant hub gene. Patients were divided into high-risk (>median risk score) and low-risk (<median risk score) groups according to the median risk score. The Kaplan–Meier method was used to estimate the survival outcomes of the high- and low-risk patients, and differences in OS were evaluated with the log-rank test. We generated a time-dependent receiver operating characteristic (ROC) curve to verify the accuracy of this signature. The classic ROC curve analysis method assumes that individual events and outcomes are fixed over time, but in practice, both disease status and outcomes change over time. Moreover, the traditional ROC curve cannot be used to analyze survival data; therefore, we adopted a time-dependent ROC curve (Kamarudin et al., 2017). *P* < 0.05 was considered statistically significant.

(1) S⁢c⁢o⁢r⁢e=∑i=1nGi⁢w*⁢e⁢i⁢g⁢h⁢ti

#### Construction and Assessment of the Nomogram

To lessen the influence of confounding factors on the relationship between gene expression and prognosis as much as possible, univariate and multivariate Cox regression analyses were performed to assess differences in clinical characteristics and risk scores. *P* < 0.05 was considered statistically significant. The nomograms of the 1-, 3-, and 5-year survival rates were constructed using the rms package in R software to visualize the prediction results^7^. The predictive ability of the nomogram was evaluated by the AUC of the ROC curve and the calibration curves for 1, 3, and 5 years.

## Results

### Correlation of the mRNAsi and Clinical Characteristics in Patients With LUSC

**FIGURE 1:**
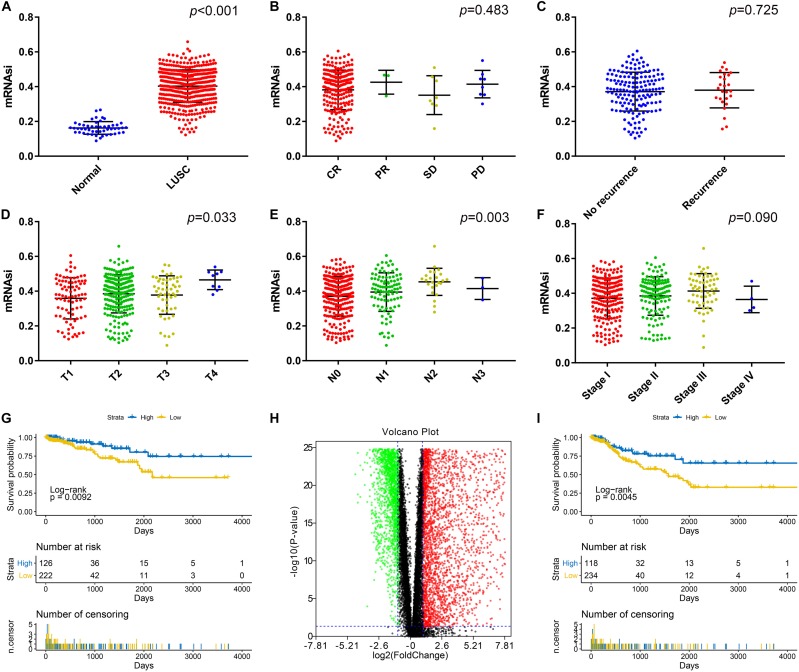
**(A)** Differences in the mRNAsi between normal (50 samples) and LUSC (487 samples) tissues. **(B)** Differences in the mRNAsi based on the treatment effect: CR (234 samples), PR (3 samples), SD (8 samples), and PD (8 samples). **(C)** Differences in the mRNAsi between LUAD patients without (159 samples) and with (26 samples) recurrence after primary treatment without adjuvant therapy. **(D)** Comparison of the mRNAsi between four different T stages (T1, 86 samples; T2, 221 samples; T3, 52 samples; and T4, 9 samples). **(E)** Comparison of the mRNAsi between four different N stages (N0, 254 samples; N1, 99 samples; N2, 24 samples; and N3, 3 samples). **(F)** Comparison of the mRNAsi between four different AJCC stages (stage I; 201 samples, stage II; 132 samples, stage III; 58 samples, and stage IV; 4 samples). **(G)** Kaplan–Meier survival curves show that the low mRNAsi group had a better OS rate than the high mRNAsi group. **(H)** Volcano map of DEGs: green indicates downregulated genes, and red indicates upregulated genes. **(I)** Kaplan–Meier curves show that the low mRNAsi group had a better PFS rate than the high mRNAsi group. LUSC, lung squamous cell carcinoma; mRNAsi, mRNA expression-based stemness index; AJCC, American Joint Committee on Cancer; OS, overall survival; PFS, progression-free survival. CR, complete response, PR, partial response, SD, stable disease, PD, progressive disease.

Ninety-two samples without enough clinical information and 12 samples without mRNAsi information from the TCGA database were excluded. As shown in Figure 1A, there was a significant difference between the mRNAsi of LUSC and normal tissues. The mRNAsi of tumor tissues was higher than that of normal tissues. Significant differences in T stage (Figure 1D) and N stage (Figure 1E) were also observed in addition to AJCC stage (Figure 1F). However, there was no significant difference in the mRNAsi based on the treatment effect (Figure 1B) or whether the patient had relapsed (Figure 1C). LUSC patients with a high mRNAsi had significantly worse OS and PFS rates than those with a low mRNAsi (Figures 1G,I).

### Screening of DEGs

There was a significant difference between the mRNAsi in normal tissues and LUSC tissues; therefore, we aimed to identify DEGs based on the comparison between the two. After log2 processing of the data, we found a total of 6122 DEGs, including 3427 upregulated genes and 2695 downregulated genes. The volcano map is shown in Figure 1H.

### WGCNA: Identification of the Most Significant Modules and Genes

**FIGURE 2:**
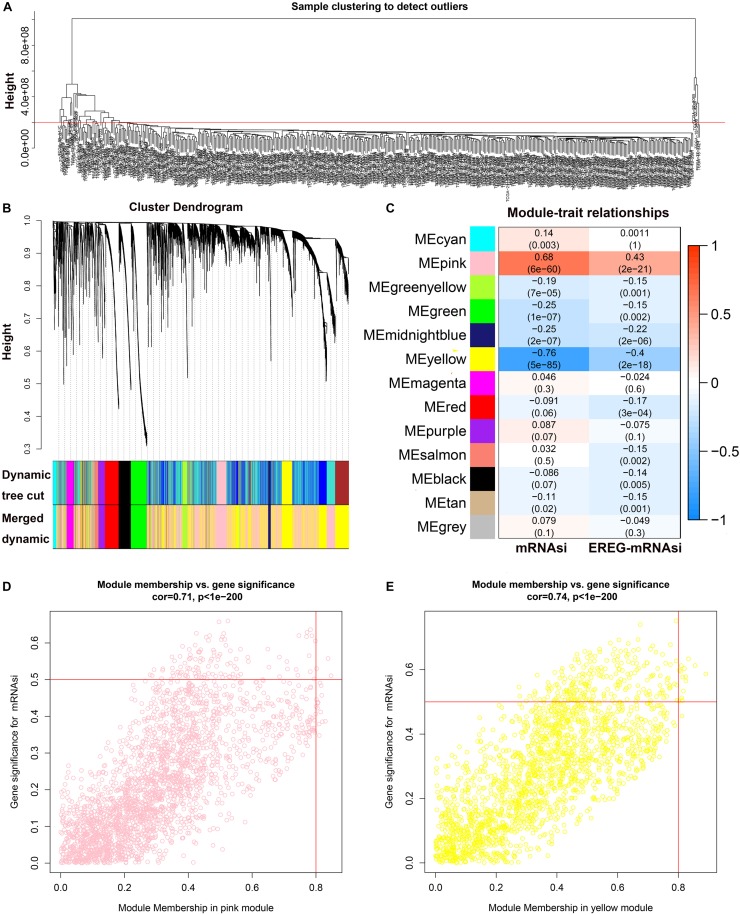
WGCNA of LUSC. **(A)** Clustering of samples and removal of outliers. **(B)** Cluster dendrogram of genes in LUSC patients. Each branch in the figure represents one gene, and each color represents one coexpression module. **(C)** Correlation between the gene module and clinical characteristics, including the mRNAsi and EREG-mRNAsi. The correlation coefficient in each cell represents the correlation between the gene module and clinical characteristics and decreases in size, from red to blue. **(D)** Scatter diagram for MM vs. GS for the mRNAsi in the pink module. **(E)** Scatter diagram for MM vs. GS for the mRNAsi in the yellow module. LUSC, lung squamous cell carcinoma; mRNAsi, mRNA expression-based stemness index; EREG, epigenetically regulated.

The results of WGCNA was shown in Supplementary Table S1. All DEGs were included in the coexpression network after excluding 48 outlier samples (Figure 2A). β = 3 met the soft-threshold parameter of the construction requirements for scale-free distribution, and the curve reached *R*^2^ = 0.925. MEDissThres was set as 0.55 to merge the similar modules, and 13 modules were ultimately obtained (Figure 2B). After the modules were evaluated for their associations with the traits of LUSC and the patient’s mRNAsi and EREG-mRNAsi, the pink (*R*^2^ = 0.68, *P* = 6e−60) module was found to be positively correlated with the mRNAsi of LUSC patients (Figure 2C), while the yellow module (*R*^2^ = −0.76, *P* = 5e−58) was found to be negatively correlated with the mRNAsi of LUSC patients (Figure 2C). In addition, the genes in the pink (cor = 0.71, *P* < 1e−200) and yellow (cor = 0.74, *P* < 1e−200) modules were characterized by high GS and MM based on an intramodular analysis (Figures 2D,E). Therefore, we chose the pink module as the most significant module for subsequent research because it showed the highest positive correlation. Based on the threshold for key genes (MM > 0.6 and cor GS > 0.4), we ultimately obtained 10 hub genes.

### Functional Enrichment Analysis

**FIGURE 3:**
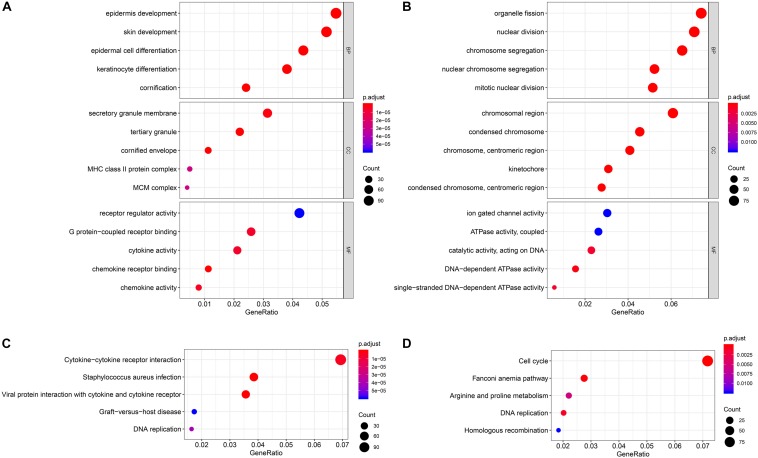
Enrichment analyses of the significant module. **(A)** GO enrichment analysis of the pink module. **(B)** GO enrichment analysis of the yellow module. **(C)** KEGG pathway enrichment analysis of the pink module. **(D)** KEGG pathway enrichment analysis of the yellow module. GO, Gene Ontology; KEGG, Kyoto Encyclopedia of Genes and Genomes.

For the two modules that were most closely related to the mRNAsi, GO and KEGG pathway enrichment analyses were performed, and the top 5 enriched results are presented in Figure 3. The pink module, which exhibited the strongest positive correlation with the mRNAsi, is highly enriched in epidermal development, the secretory granule membrane, receptor regulator activity and the cytokine–cytokine receptor interaction, while the yellow module, which exhibited the strongest negative correlation with the mRNAsi, is highly enriched in organelle fission, the chromosomal region, ion-gated channel activity and the cell cycle.

### Protein-Protein Interaction (PPI) Network and Hub Gene Validation

**FIGURE 4:**
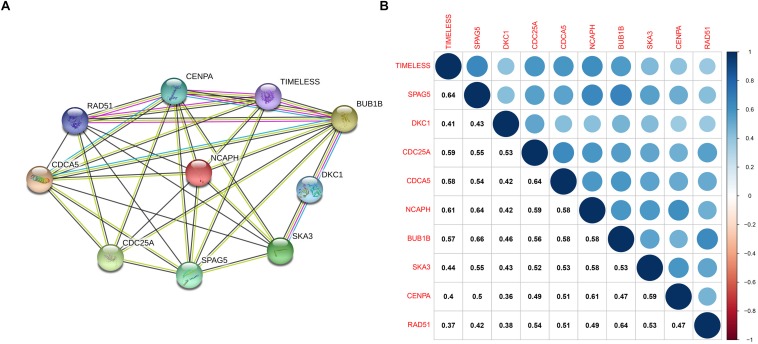
**(A)** Protein–protein interactions between hub genes. The thickness of the solid line represents the strength of the relationship. **(B)** Correlation between the hub genes.

**FIGURE 5:**
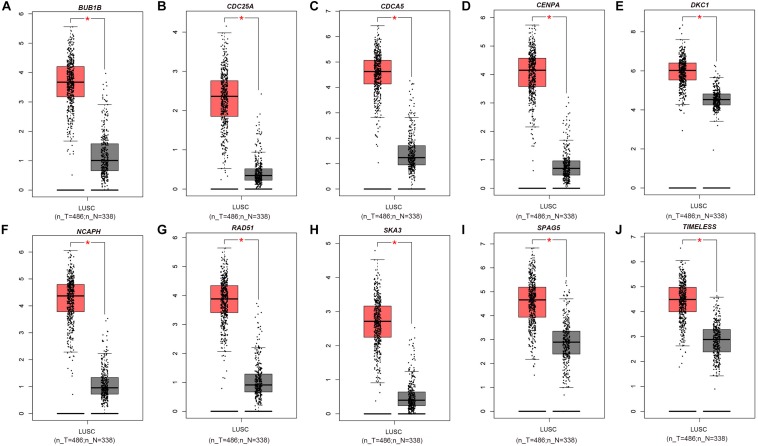
Box plot of the top 10 hub genes whose expression levels were verified with data from the GEPIA database: **(A)** *BUB1B*, **(B)** *CDC25A*, **(C)** *CDCA5*, **(D)** *CENPA*, **(E)** *DKC1*, **(F)** *NCAPH*, **(G)** *RAD51*, **(H)** *SKA3*, **(I)** *SPAG5*, and **(J)** *TIMELESS*. Red * indicates *P* < 0.05.

**FIGURE 6:**
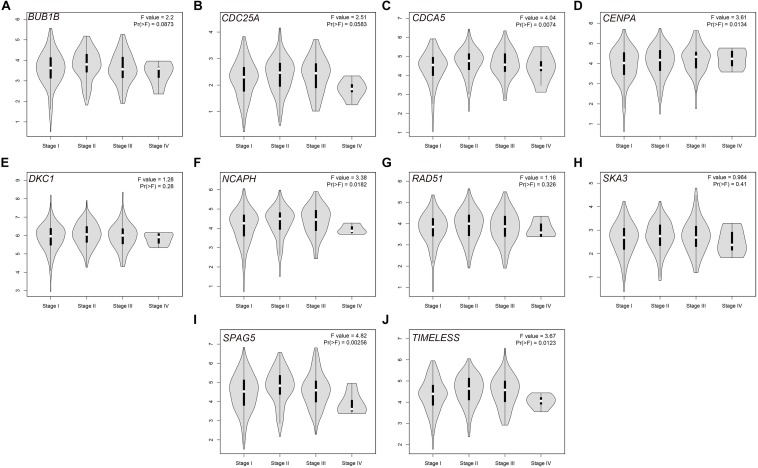
Violin plots of the expression levels of the selected hub genes in different stages via the GEPIA database: **(A)** *BUB1B*, **(B)** *CDC25A*, **(C)** *CDCA5*, **(D)** *CENPA*, **(E)** *DKC1*, **(F)** *NCAPH*, **(G)** *RAD51*, **(H)** *SKA3*, **(I)** *SPAG5*, and **(J)** *TIMELESS*.

The PPI network, consisting of the top 10 hub genes, was constructed using the STRING database. In total, 10 nodes and 33 edges were included in this PPI network (Figure 4A), with an average node degree of 6.6 and strong correlations. The 10 hub genes were also significantly correlated with each other at the transcriptional level (Figure 4B). The expression levels of the top 10 hub genes were higher in tumor tissue than in normal tissue (Figure 5). However, only 5 hub genes were differentially expressed in different TNM stages (Figure 6): *CDCA5, CENPA, NCAPH, SPAG5*, and *TIMELESS*.

### Survival Analysis

#### Establishment of the Prognostic Signature

**FIGURE 7:**
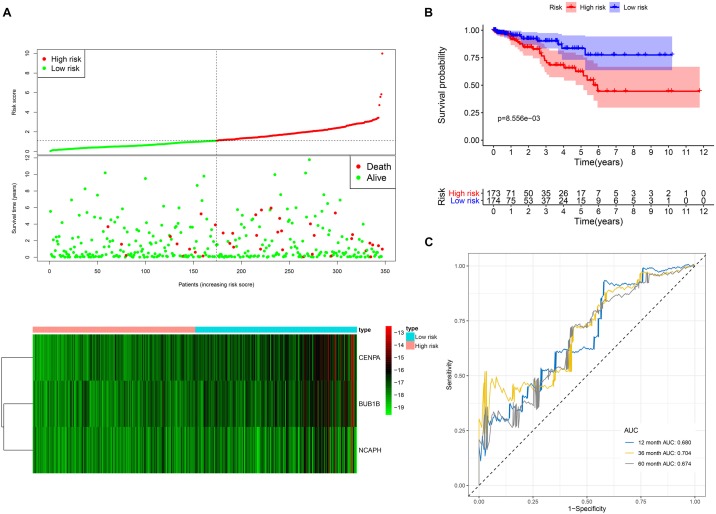
The three-hub mRNA signature used to predict OS in LUSC patients. **(A)** Distribution, patient survival status and heatmap of the three-hub mRNA expression profiles. **(B)** Kaplan–Meier survival estimates of OS in LUSC patients according to the three-hub mRNA signature. **(C)** ROC analysis for the prediction of 1-, 3-, and 5-year OS as the defining point of the three-hub mRNA signature. LUSC, lung squamous cell carcinoma; ROC, receiver operating characteristic; OS, overall survival.

The prognostic signature consists of three hub genes (Figure 7A) that were incorporated into the multivariate Cox proportional hazards regression analysis to obtain the coefficients of the three hub genes that were used in Equation 1 to calculate the risk scores (Table 1). The risk score was calculated as follows: (−0.630^∗^ expression level of BUB1B) + (−0.652^∗^ expression level of CENPA) + (2.163^∗^ expression level of NCAPH) (Table 1). The prognosis of high-risk patients was significantly worse than that of low-risk patients (Figure 7B). The AUC values of 1-, 3-, and 5-year OS were 0.680, 0.704, and 0.674, respectively (Figure 7C).

**TABLE 1:** Multivariable Cox regression analysis of the three-hub gene signature.

**Gene**	**Univariate analysis**			**Multivariate analysis**			**Coefficient**
	**HR**	**95%CI**	***P*-value**	**HR**	**95%CI**	***P*-value**	
BUB1B	0.642	0.425–0.970	0.035	0.543	0.233–0.764	0.008	−0.630
CDC25A	0.757	0.521–0.972	0.046	1.028	0.484–2.184	0.940	–
CDCA5	0.797	0.495–1.238	0.350				
CENPA	0.665	0.449–0.985	0.042	0.498	0.244–0.952	0.046	−0.652
DKC1	0.607	0.352–1.048	0.073				–
NCAPH	1.313	1.172–2.213	0.006	2.286	1.172–4.213	0.023	2.163
RAD51	0.719	0.454–1.138	0.159				–
SKA3	0.781	0.512–1.191	0.025	1.346	0.693–2.617	0.380	–
SPAG5	0.685	0.474–0.990	0.044	0.604	0.313–1.169	0.134	–
TIMELESS	1.049	1.020–2.418	0.029	1.049	0.455–2.418	0.904	–

#### Construction and Assessment of the Nomogram

**FIGURE 8:**
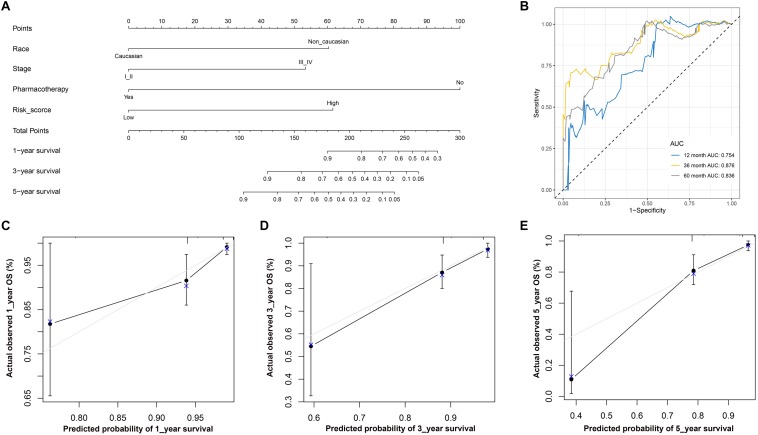
**(A)** Nomogram used to predict 1-, 3-, and 5-year OS. **(B)** ROC curve based on the nomogram for 1-, 3-, and 5-year OS probability. **(B)** ROC analysis of 1-, 3-, and 5-year OS as the defining points of the nomogram. **(C)** Calibration curves for predicting 1–year OS. The nomogram–predicted probability of survival is plotted on the *x*–axis; actual survival is plotted on the *y*–axis. **(D)** Calibration curves for predicting 3–year OS. **(E)** Calibration curves for predicting 5–year OS. According to the scores of the corresponding variables in the nomogram, namely, the points at the top of the chart, the 1-, 3-, and 5-year survival rates of patients can be predicted according to the total scores of all corresponding individual scores. Theoretically, the standard curve is a straight line with a slope of 1 through the origin of the coordinate axis. If the predicted calibration curve is closer to the standard curve, the better the prediction capacity of the nomogram is. LUSC, lung squamous cell carcinoma; ROC, receiver operating characteristic; OS, overall survival.

The nomogram included the independent risk factors identified by the univariate and multivariate Cox regression analyses (Table 2): race (Caucasian vs. non-Caucasian), AJCC stage (stages I-II vs. stages III-IV), pharmacotherapy (no vs. yes) and risk score (low vs. high). The nomogram was constructed with these risk factors (Figure 8A). The AUC values for 1-, 3-, and 5-year OS were 0.754, 0.876 and 0.836 (Figure 8B), respectively. The calibration curve also demonstrated good capacity for the nomogram to predict 1- (Figure 8C), 3- (Figure 8D), and 5- (Figure 8E) year OS. The code for R software in this study can be obtained from Supplementary Data Sheet S1.

**TABLE 2:** Univariable and multivariable Cox regression analyses of clinical characteristics.

**Variable**	**Univariate analysis**			**Multivariate analysis**		
	**HR**	**95%CI**	***P*-value**	**HR**	**95%CI**	***P*-value**
Age	1.060	1.011–1.111	0.015	1.036	0.990–1.083	0.128
Sex (Female/Male)	0.878	0.442–1.745	0.711	–		
Race (Non-Caucasian/Caucasian)	0.274	0.107–0.703	0.007	0.231	0.086–0.609	0.003
T stage (T1-T2/T3-T4)	1.449	0.603–3.485	0.407	–		
N stage (N0-N1/N2-N3)	1.577	0.612–4.068	0.346	–		
M stage (M0/M1)	1.674	0.843–3.271	0.670	–		
AJCC stage (I-II/III-IV)	1.588	1.079–3.423	0.027	3.628	1.539–8.549	0.006
Pharmacotherapy (No/Yes)	0.095	0.013–0.696	0.020	0.072	0.009–0.552	0.011
Radiotherapy (No/Yes)	0.737	0.226–2.400	0.612	–		
Risk score (Low/High)	6.616	2.877–15.212	<0.001	4.517	1.956–10.434	<0.001

## Discussion

LUSC is associated with extremely high mortality and morbidity, but its pathogenesis is still unclear. However, an increasing number of studies have found that cancer stem cells (CSCs) play an important role in the development and drug resistance of NSCLC (Herreros-Pomares et al., 2019; Huang et al., 2019; Tahmasebi et al., 2019). In this study, we identified the significance of the mRNAsi in the clinical characteristics of patients with LUSC with the help of data from the TCGA database and the mRNAsi corresponding to each sample. Moreover, hub genes related to the mRNAsi were obtained by the WGCNA method, and the association between the change in hub gene expression and clinical features was verified by external data from the GEPIA database. The results also indicated that all hub genes are highly expressed in tumor tissues, and some hub genes are of great significance as the disease progresses. Finally, after adjusting for the effects of confounding factors, we obtained a prognostic signature containing three prognostic genes with great predictive capacity. Two hub genes were not only highly expressed in LUSC but also associated with TNM stage and prognosis: *CENPA* and *NCAPH*.

*CENPA* (centromere protein A) is the key determinant of centromere identity (Fujita et al., 2015). The centromere is the basis of centromere formation and the key to chromosome separation during mitosis. *CENPA* controls the epigenetic identity of the centromere, which is essential for recruiting centromere elements to connect chromosomes to the mitotic spindle during mitosis (Rošic and Erhardt, 2016). Behnan et al. (2016) showed that the inhibition of *CENPA* expression in glioblastoma cells can reduce sphere-forming capacity, proliferation, and cell viability. *In vitro* experiments by Cheng et al. (2019) in mice confirmed that elevated *FOXM1* expression enhanced *CENPA* and *CENPB* expression, which promoted cell cycle progression and cell proliferation, thereby promoting LUSC cell growth. Regarding prognosis, lung adenocarcinoma (LUAD) patients with high *CENPA* expression experience poor OS based on data integrated from six different GEO chips by Liu et al. (2018).

*NCAPH* (non-SMC condensin I complex subunit H), whose expression is significantly high in both LUAD and LUSC (Ma et al., 2019), belongs to the protein superfamily defined as kleisins (Neuwald and Hirano, 2000). *NCAPH* plays an important role in the separation of the cell’s sister chromatids and the maintenance of the mitotic chromosomal structure, and studies have demonstrated that its biallelic mutation can lead to a significant reduction in brain size in mice (Martin et al., 2016). Gene knockout experiments in mice by Sun et al. (2019) confirmed that *NCAPH* promotes the proliferation, migration and invasion of liver cancer *in vivo* and *in vitro*. The same experimental method also indicated that *NCAPH* is a potential candidate for radiation tolerance (Wang X.C. et al., 2019). High *NCAPH* expression also suggests a poor prognosis in prostate (Cui et al., 2019) and rectal (Yin et al., 2017) cancer patients.

*BUB1B* (BUB1 mitotic checkpoint serine/threonine kinase B) encodes a kinase involved in spindle checkpoint function and mitosis and plays an important role in the development of many types of cancer (Baker et al., 2013). Interestingly, *BUB1B* is also highly expressed in SCLC. For patients with SCLC, the higher the expression level of *BUB1B* is, the worse the prognosis (Liao et al., 2019). *BUB1B* has also been found to regulate the development of stem cells; for example, in experiments with embryonic stem cells, Su et al. (2019) found that knocking down *BUB1B* can lead to DNA damage and other forms of genomic instability, activate p53 and eventually lead to embryonic stem cell differentiation and possibly cancer.

*CDC25A* (cell division cycle 25A) and *CDCA5* (cell division cycle-associated 5) are important regulators of cell mitosis. *CDC25A* is a “switch” protein that controls G1/S and G2/M checkpoints and plays an important role in maintaining the stability of DNA replication and the integrity of the cell division cycle (Russell and Nurse, 1986). *CDCA5* is located on human chromosome 11q13.1, and its function is to ensure accurate separation of the sister chromosome during anaphase mitosis (Schmitz et al., 2007). *CDC25A* is overexpressed in many tumor cells and is associated with the malignancy and prognosis of several cancers, such as human glioma (Yamashita et al., 2010), retinoblastoma (Singh et al., 2015), breast cancer (Brunetto et al., 2013), and liver cancer (Lu et al., 2016). *CDCA5* is highly expressed in NSCLC (Chang et al., 2015) and liver cancer (Shen et al., 2018) and is associated with a poor prognosis. The genes in the CDC family are key in driving the cell cycle, as well as promoting the formation of centrosomes and mitotic spindles. *CDCA5* is also a biomarker of malignant glioma of neural stem cell origin (Hembram et al., 2019).

*DKC1* (dyskerin pseudouridine synthase 1) is highly conserved and widely expressed and may play additional roles in nucleocytoplasmic shuttling, the DNA damage response, and cell adhesion. The *DKC1* gene encodes a protein responsible for the stability of the telomerase whole enzyme complex. The mutation of *DKC1* has a strong influence on telomere repair and hematopoietic development. Induced pluripotent stem cells extracted from the fibroblasts of patients with X-linked keratosis disorder demonstrated that defective *DKC1* count not extend telomeres (Donaires et al., 2019). A study by Nersisyan et al. (2019) found that *DKC1* may also be involved in the activation of telomere maintenance mechanisms that lead to cancer. *DKC1* downregulation can also inhibit the growth of glioma cells by altering the expression of cell cycle-related molecules, causing cells to arrest in G1 phase. *In vitro* experiments also confirmed that glioma cells with *DKC1* knockout showed low activity (Miao et al., 2019). *DKC1* is highly expressed in clear cell renal cell carcinoma. *DKC1* knockout inhibits tumor proliferation, migration, and invasion by regulating the NF-κB/MMP-2 signaling pathway *in vitro* (Zhang M. et al., 2018).

*RAD51* (RAD51 recombinase) is a key player in homologous recombination because it is closely related to and binds to DNA and exhibits ATPase activity following stimulation (Zhang et al., 2009). Chiu et al. (2019) found that the overexpression of *RAD51* promotes the viability of esophageal cancer cells, while its inhibition weakens the viability of esophageal cancer cells through cell cycle entry, migration/invasion and epithelial-mesenchymal transformation. Another study also found a mutation in the stem cell marker *RAD51* upon exon sequencing in patients with esophageal squamous cell carcinoma (ESCC) and revealed that *RAD51* is related to the drug sensitivity of ESCC (Golyan et al., 2020). High *RAD51* expression enhances cancer progression through the p38/Akt/Snail signaling pathway (Chiu et al., 2019). *RAD51* may lead to increased drug resistance in triple-negative breast cancer patients (Zhao et al., 2019) and is also associated with the radiosensitivity of some tumors, such as nasopharyngeal cancer (Zhang Z. et al., 2018) and prostate cancer (Maranto et al., 2018).

Three other genes also merit further study. *SKA3* (spindle and kinetochore-associated complex subunit 3) is an important subunit of the spindle and centromere-related protein complex and plays a regulatory role in cell proliferation and apoptosis (Zhang et al., 2017). A previous study showed that the incidence of age-related neurodegenerative diseases is consistent with a dramatic decline in the number and function of adult neural stem cells, while *SKA3* is closely associated with age-related central nervous system diseases. *SKA3* overexpression promotes the growth and migration of cervical cancer cells by activating the PI3K-AKT signaling pathway and promoting cell cycle progression (Hu et al., 2018). *SPAG5* (sperm-associated antigen 5) binds to microtubules and centromeres of the spindle during mitosis or meiosis as a spindle-binding protein (Cheng et al., 2007). A previous study showed that *SPAG5* inhibits apoptosis by activating mammalian target of rapamycin 1 (mTORC1) (Thedieck et al., 2013). *SPAG5* has also been linked to the development and metastasis of LUAD (Wang T. et al., 2019), stomach cancer (Liu et al., 2019), and breast cancer (Jiang et al., 2019).

*TIMELESS* (timeless circadian regulator) is one of the core genes involved in the biological rhythm and is abnormally expressed in liver cancer, breast cancer and lung cancer (Truong et al., 2016; Shostak, 2017). In recent years, it has been found that the expression of *TIMELESS* in tumors is not completely uniform: it is weakly expressed in renal cell carcinoma, ductal cell carcinoma of the pancreas and other malignant tumors and is closely related to poor patient survival. However, it is highly expressed in colon cancer and significantly correlated with lymphatic metastasis, satellite metastasis and TNM stage (Top et al., 2016; Ozturk et al., 2017). *TIMELESS* plays a crucial role in the self-renewal process of breast CSCs and interacts with Sp1/c-jun to induce miR-5188 expression by promoting c-jun-mediated transcription, thus promoting breast cancer progression (Zou et al., 2020).

Our research also has the following limitations. First, we used data from a public database to confirm our findings and did not perform further experiments to confirm the expression of related genes or the molecular mechanisms and pathways involved. Second, since our study examined data from a public database and online tools, the quality of these data may not be guaranteed. Finally, the data we studied were obtained almost entirely from the United States and are not representative of patients worldwide. Therefore, further well-designed biological studies with large sample sizes are needed to confirm our findings.

## Conclusion

*CDC25A, DKC1, CDCA5, BUB1B, SKA3, TIMELESS, NCAPH, SPAG5, CENPA*, and *RAD51* may have a strong influence on LUSC stem cell maintenance. These hub genes may serve as control targets for LUSC CSCs, and further study of these genes may lead to new anticancer therapies.

## Data Availability Statement

RNA-seq and clinical information for each sample were downloaded from the TCGA website (https://cancergenome. nih.gov/).

## Author Contributions

YL conceived and designed the study, acquired and analyzed the data, and wrote the manuscript. HX and MC contributed to data analysis and manuscript drafting. XF designed the study and revised the manuscript.

## Conflict of Interest

The authors declare that the research was conducted in the absence of any commercial or financial relationships that could be construed as a potential conflict of interest.

## Footnotes

1 https://cancergenome.nih.gov/

2 http://asia.ensembl.org/index.html

3 https://string-db.org/

4 https://cran.r-project.org/web/packages/corrplot/index.html

5 http://gepia.cancer-pku.cn/

6 https://www.gtexportal.org/

7 http://CRAN.Rproject.org/packagerms

## Supplementary Material

FIGURE S1 A flow chart of the research conducted.

TABLE S1 The results of WGCNA.

DATA SHEET S1 The code of R software.
