# Supplementary figures and images for: Bioinformatics Analysis Reveals Biomarkers With Cancer Stem Cell Characteristics in Lung Squamous Cell Carcinoma
Source: Front Genet. 2020 May 13;11:427. doi: 10.3389/fgene.2020.00427 (PMC7247832; doi:10.3389/fgene.2020.00427)

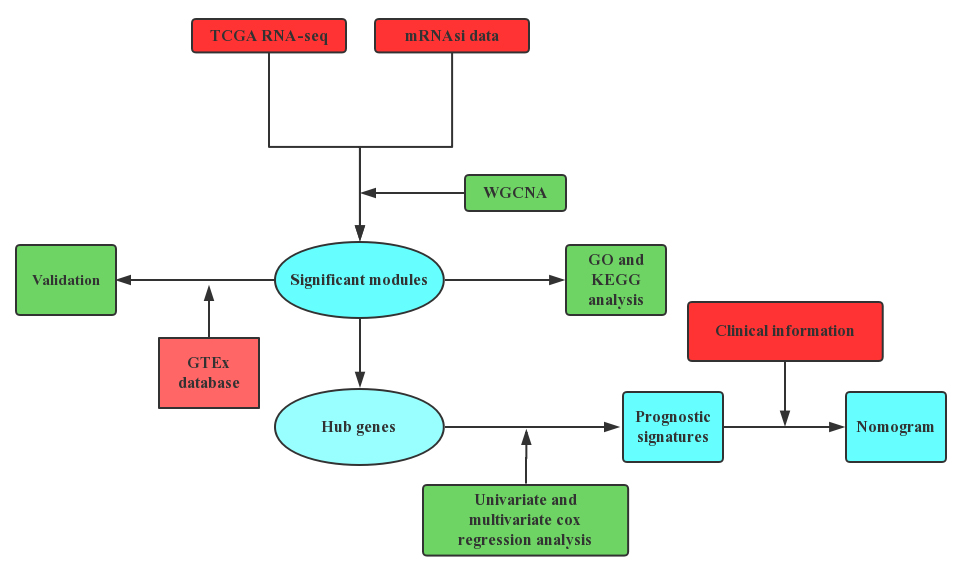

Supplement: FIGURE S1 — A flow chart of the research conducted. [file Image_1.JPEG]
