# Supplementary material for: Bioinformatics Analysis Reveals Biomarkers With Cancer Stem Cell Characteristics in Lung Squamous Cell Carcinoma
Source: Front Genet. 2020 May 13;11:427. doi: 10.3389/fgene.2020.00427 (PMC7247832; doi:10.3389/fgene.2020.00427)
Supplement: DATA SHEET S1 — The code of R software. [file Data_Sheet_1.PDF]

**The following code can be copied directly to run in R software, and the content after # will not run in R software.**

**DEGs**

```
library("limma")

setwd("C:\\Users\\Administrator\\Desktop\\lusc\\2diff")                    #Set up the working
directory
inputFile="symbol.txt"                                                         #The input
file
fdrFilter=0.05                                                                 #false
discovery rate
logFCfilter=1                                                      #logFC (fold change)
conNum=50
#Number of samples of normal group
treatNum=487
#Sample number of LUSC group

#Read input file
outTab=data.frame()
grade=c(rep(1,conNum),rep(2,treatNum))
rt=read.table(inputFile,sep="\t",header=T,check.names=F)
rt=as.matrix(rt)
rownames(rt)=rt[,1]
exp=rt[,2:ncol(rt)]
dimnames=list(rownames(exp),colnames(exp))
data=matrix(as.numeric(as.matrix(exp)),nrow=nrow(exp),dimnames=dimnames)
data=avereps(data)
data=data[rowMeans(data)>0.2,]

#Variance analysis
for(i in row.names(data)){
  geneName=unlist(strsplit(i,"\\|",))[1]
  geneName=gsub("\\/", "_", geneName)
  rt=rbind(expression=data[i,],grade=grade)
  rt=as.matrix(t(rt))
  wilcoxTest<-wilcox.test(expression ~ grade, data=rt)
  conGeneMeans=mean(data[i,1:conNum])
  treatGeneMeans=mean(data[i,(conNum+1):ncol(data)])
  logFC=log2(treatGeneMeans)-log2(conGeneMeans)
  pvalue=wilcoxTest$p.value
  conMed=median(data[i,1:conNum])
```

```
    treatMed=median(data[i,(conNum+1):ncol(data)])
    diffMed=treatMed-conMed
      if( ((logFC>0) & (diffMed>0)) | ((logFC<0) & (diffMed<0)) ){

outTab=rbind(outTab,cbind(gene=i,conMean=conGeneMeans,treatMean=treatGeneMean
s,logFC=logFC,pValue=pvalue))
        }
}
pValue=outTab[,"pValue"]
fdr=p.adjust(as.numeric(as.vector(pValue)),method="fdr")
outTab=cbind(outTab,fdr=fdr)

#Output the differences of all genes
write.table(outTab,file="all.xls",sep="\t",row.names=F,quote=F)

#Output difference table
outDiff=outTab[( abs(as.numeric(as.vector(outTab$logFC)))>logFCfilter &
as.numeric(as.vector(outTab$fdr))<fdrFilter),]
write.table(outDiff,file="diff.xls",sep="\t",row.names=F,quote=F)

#The expression of the output difference
heatmap=rbind(ID=colnames(data[as.vector(outDiff[,1]),]),data[as.vector(outDiff[,1]),])
write.table(heatmap,file="diffGeneExp.txt",sep="\t",col.names=F,quote=F)
```

WGCNA

```
library("WGCNA")                              #Reference WGCNA package
setwd("C:\\Users\\Administrator\\Desktop\\lusc\\3.wgcna")
#Set up the working directory
data=read.table("diffGeneExp.txt",sep="\t",header=T,check.names=F,row.names=1)
#Read expression file

#Delete the normal sample,If not, skip this step
group=sapply(strsplit(colnames(data),"\\-"),"[",4)
group=sapply(strsplit(group,""),"[",1)
group=gsub("2","1",group)
data=data[,group==0]
datExpr0=t(data)
```

```
###Check for missing values
gsg = goodSamplesGenes(datExpr0, verbose = 3)
if (!gsg$allOK)
{
  # Optionally, print the gene and sample names that were removed:
  if (sum(!gsg$goodGenes)>0)
    printFlush(paste("Removing genes:", paste(names(datExpr0)[!gsg$goodGenes],
collapse = ", ")))
  if (sum(!gsg$goodSamples)>0)
    printFlush(paste("Removing samples:",
paste(rownames(datExpr0)[!gsg$goodSamples], collapse = ", ")))
  # Remove the offending genes and samples from the data:
  datExpr0 = datExpr0[gsg$goodSamples, gsg$goodGenes]
}

###The sample clustering
sampleTree = hclust(dist(datExpr0), method = "average")
pdf(file = "1_sample_cluster.pdf", width = 17, height = 7)
par(cex = 0.6)
par(mar = c(0,4,2,0))
plot(sampleTree, main = "Sample clustering to detect outliers", sub="", xlab="", cex.lab
= 1.5, cex.axis = 1.5, cex.main = 2)
###Shear line
abline(h = 200000000, col = "red")
dev.off()

###Delete the sample below the shear line
clust = cutreeStatic(sampleTree, cutHeight = 200000000, minSize = 10)
table(clust)
keepSamples = (clust==1)
datExpr0 = datExpr0[keepSamples, ]


###Read the mRNAsi data
traitData = read.table("mRNAsi.txt",row.names=1,header=T,comment.char =
"",check.names=F)
fpkmSamples = rownames(datExpr0)
traitSamples =rownames(traitData)
sameSample=intersect(fpkmSamples,traitSamples)
datExpr0=datExpr0[sameSample,]
datTraits=traitData[sameSample,]
```

```
###The sample clustering
sampleTree2 = hclust(dist(datExpr0), method = "average")
traitColors = numbers2colors(datTraits, signed = FALSE)
pdf(file="2_sample_heatmap.pdf",width=12,height=17)
plotDendroAndColors(sampleTree2, traitColors,
                    groupLabels = names(datTraits),
                    main = "Sample dendrogram and trait heatmap")
dev.off()

###Scatter plots of power values
enableWGCNAThreads()    #Multithreaded work
powers = c(1:20)        #The power range is 1:20
sft = pickSoftThreshold(datExpr0, powerVector = powers, verbose = 5)
pdf(file="3_scale_independence.pdf",width=9,height=8)
par(mfrow = c(1,2))
cex1 = 0.9
###Fit the scatter plot of exponential and power value
plot(sft$fitIndices[,1], -sign(sft$fitIndices[,3])*sft$fitIndices[,2],
     xlab="Soft Threshold (power)",ylab="Scale Free Topology Model Fit,signed
R^2",type="n",
     main = paste("Scale independence"));
text(sft$fitIndices[,1], -sign(sft$fitIndices[,3])*sft$fitIndices[,2],
     labels=powers,cex=cex1,col="red");
abline(h=0.90,col="red") #You can modify it
###Scatter plots of average connectivity and power values
plot(sft$fitIndices[,1], sft$fitIndices[,5],
     xlab="Soft Threshold (power)",ylab="Mean Connectivity", type="n",
     main = paste("Mean connectivity"))
text(sft$fitIndices[,1], sft$fitIndices[,5], labels=powers, cex=cex1,col="red")
dev.off()


###Adjacency matrix transformation
sft #See the best power values
softPower =sft$powerEstimate #The best power value
adjacency = adjacency(datExpr0, power = softPower)

###TOM matrix
TOM = TOMsimilarity(adjacency);
```

```
dissTOM = 1-TOM

###Genetic clustering
geneTree = hclust(as.dist(dissTOM), method = "average");
pdf(file="4_gene_clustering.pdf",width=12,height=9)
plot(geneTree, xlab="", sub="", main = "Gene clustering on TOM-based dissimilarity",
     labels = FALSE, hang = 0.04)
dev.off()


###Dynamic shear module recognition
minModuleSize = 50          #Number of module genes
dynamicMods = cutreeDynamic(dendro = geneTree, distM = dissTOM,
                            deepSplit = 2, pamRespectsDendro = FALSE,
                            minClusterSize = minModuleSize);
table(dynamicMods)
dynamicColors = labels2colors(dynamicMods)
table(dynamicColors)
pdf(file="5_Dynamic_Tree.pdf",width=8,height=6)
plotDendroAndColors(geneTree, dynamicColors, "Dynamic Tree Cut",
                    dendroLabels = FALSE, hang = 0.03,
                    addGuide = TRUE, guideHang = 0.05,
                    main = "Gene dendrogram and module colors")
dev.off()


###Clustering of similar modules
MEList = moduleEigengenes(datExpr0, colors = dynamicColors)
MEs = MEList$eigengenes
MEDiss = 1-cor(MEs);
METree = hclust(as.dist(MEDiss), method = "average")
pdf(file="6_Clustering_module.pdf",width=7,height=6)
plot(METree, main = "Clustering of module eigengenes",
     xlab = "", sub = "")
MEDissThres = 0.55 #The shear height can be modified
abline(h=MEDissThres, col = "red")
dev.off()


###Similar module merging
```

```
merge = mergeCloseModules(datExpr0, dynamicColors, cutHeight = MEDissThres,
verbose = 3)
mergedColors = merge$colors
mergedMEs = merge$newMEs
pdf(file="7_merged_dynamic.pdf", width = 9, height = 6)
plotDendroAndColors(geneTree, cbind(dynamicColors, mergedColors),
                    c("Dynamic Tree Cut", "Merged dynamic"),
                    dendroLabels = FALSE, hang = 0.03,
                    addGuide = TRUE, guideHang = 0.05)
dev.off()
moduleColors = mergedColors
table(moduleColors)
colorOrder = c("grey", standardColors(50))
moduleLabels = match(moduleColors, colorOrder)-1
MEs = mergedMEs


###Module and character data heat map
nGenes = ncol(datExpr0)
nSamples = nrow(datExpr0)
moduleTraitCor = cor(MEs, datTraits, use = "p")
moduleTraitPvalue = corPvalueStudent(moduleTraitCor, nSamples)
pdf(file="8_Module_trait.pdf",width=6,height=6)
textMatrix = paste(signif(moduleTraitCor, 2), "\n(",
                   signif(moduleTraitPvalue, 1), ")", sep = "")
dim(textMatrix) = dim(moduleTraitCor)
par(mar = c(5, 10, 3, 3))
labeledHeatmap(Matrix = moduleTraitCor,
               xLabels = names(datTraits),
               yLabels = names(MEs),
               ySymbols = names(MEs),
               colorLabels = FALSE,
               colors = blueWhiteRed(50),
               textMatrix = textMatrix,
               setStdMargins = FALSE,
               cex.text = 0.5,
               zlim = c(-1,1),
               main = paste("Module-trait relationships"))
dev.off()
```

```
###Calculate MM and GS values
modNames = substring(names(MEs), 3)
geneModuleMembership = as.data.frame(cor(datExpr0, MEs, use = "p"))
MMPvalue = as.data.frame(corPvalueStudent(as.matrix(geneModuleMembership),
nSamples))
names(geneModuleMembership) = paste("MM", modNames, sep="")
names(MMPvalue) = paste("p.MM", modNames, sep="")
traitNames=names(datTraits)
geneTraitSignificance = as.data.frame(cor(datExpr0, datTraits, use = "p"))
GSPvalue = as.data.frame(corPvalueStudent(as.matrix(geneTraitSignificance),
nSamples))
names(geneTraitSignificance) = paste("GS.", traitNames, sep="")
names(GSPvalue) = paste("p.GS.", traitNames, sep="")


###Batch output of miRNAsi and module scatter diagrams
for (trait in traitNames){
  traitColumn=match(trait,traitNames)
  for (module in modNames){
    column = match(module, modNames)
    moduleGenes = moduleColors==module
    if (nrow(geneModuleMembership[moduleGenes,]) > 1){
      outPdf=paste("9_", trait, "_", module,".pdf",sep="")
      pdf(file=outPdf,width=7,height=7)
      par(mfrow = c(1,1))
      verboseScatterplot(abs(geneModuleMembership[moduleGenes, column]),
                         abs(geneTraitSignificance[moduleGenes, traitColumn]),
                         xlab = paste("Module Membership in", module,
"module"),
                         ylab = paste("Gene significance for ",trait),
                         main = paste("Module membership vs. gene
significance\n"),
                         cex.main = 1.2, cex.lab = 1.2, cex.axis = 1.2, col =
module)
      abline(v=0.8,h=0.5,col="red")
      dev.off()
    }
  }
}
```

```
###Output GS_MM data
probes = colnames(datExpr0)
geneInfo0 = data.frame(probes= probes,
                       moduleColor = moduleColors)
for (Tra in 1:ncol(geneTraitSignificance))
{
  oldNames = names(geneInfo0)
  geneInfo0 = data.frame(geneInfo0, geneTraitSignificance[,Tra],
                         GSPvalue[, Tra])
  names(geneInfo0) = c(oldNames,names(geneTraitSignificance)[Tra],
                       names(GSPvalue)[Tra])
}

for (mod in 1:ncol(geneModuleMembership))
{
  oldNames = names(geneInfo0)
  geneInfo0 = data.frame(geneInfo0, geneModuleMembership[,mod],
                         MMPvalue[, mod])
  names(geneInfo0) = c(oldNames,names(geneModuleMembership)[mod],
                       names(MMPvalue)[mod])
}
geneOrder =order(geneInfo0$moduleColor)
geneInfo = geneInfo0[geneOrder, ]
write.table(geneInfo, file = "GS_MM.xls",sep="\t",row.names=F)
```

**GO and KEGG**

```
setwd("C:\\Users\\Administrator\\Desktop\\lusc\\4.go\\pink\\09.symbo2id")
#Set up the working directory

library("org.Hs.eg.db")          #Reference the package
rt=read.table("symbol.txt",sep="\t",check.names=F,header=T)      #Read the file
genes=as.vector(rt[,1])
entrezIDs <- mget(genes, org.Hs.egSYMBOL2EG, ifnotfound=NA)      #Find the id for
the gene
entrezIDs <- as.character(entrezIDs)
out=cbind(rt,entrezID=entrezIDs)
write.table(out,file="id.txt",sep="\t",quote=F,row.names=F)      #Output result

library("clusterProfiler")
library("org.Hs.eg.db")
```

```
library("enrichplot")
library("ggplot2")

setwd("C:\\Users\\Administrator\\Desktop\\lusc\\4.go\\yellow\\10.GO")
#Set up the working directory
rt=read.table("id.txt",sep="\t",header=T,check.names=F)                    #Read the id.txt
file
rt=rt[is.na(rt[,"entrezID"])==F,]                                            #Remove the
gene whose id is NA
gene=rt$entrezID

#GO analysis
kk <- enrichGO(gene = gene,
               OrgDb = org.Hs.eg.db,
               pvalueCutoff =0.05,
               qvalueCutoff = 0.05,
               ont="all",
               readable =T)
write.table(kk,file="GO.txt",sep="\t",quote=F,row.names = F)
#Preserved enrichment results

#A histogram
pdf(file="barplot.pdf",width = 10,height = 8)
barplot(kk, drop = TRUE, showCategory =10,split="ONTOLOGY") +
facet_grid(ONTOLOGY~., scale='free')
dev.off()

#Bubble chart
pdf(file="bubble.pdf",width = 8,height = 3)
dotplot(kk,showCategory = 5,split="ONTOLOGY") + facet_grid(ONTOLOGY~.,
scale='free')
dev.off()

library("clusterProfiler")
library("org.Hs.eg.db")
library("enrichplot")
library("ggplot2")

setwd("C:\\Users\\Administrator\\Desktop\\lusc\\4.go\\pink\\12.KEGG")
#Set up the working directory
rt=read.table("id.txt",sep="\t",header=T,check.names=F)          #Read the id.txt file
```

```
rt=rt[is.na(rt[,"entrezID"])==F,]                                          #Remove the gene
whose id is NA
gene=rt$entrezID

#KEGG analysis
kk <- enrichKEGG(gene = gene, organism = "hsa", pvalueCutoff =0.05, qvalueCutoff
=0.05)     #Analysis of enrichment
write.table(kk,file="KEGGId.txt",sep="\t",quote=F,row.names = F)
#Preserved enrichment results

#A histogram
pdf(file="barplot.pdf",width = 10,height = 7)
barplot(kk, drop = TRUE, showCategory = 30)
dev.off()

#Bubble chart
pdf(file="bubble.pdf",width = 9.5,height = 3)
dotplot(kk, showCategory = 5)
dev.off()
```

Survival analysis

```
library(survival)
library(survminer)
setwd("C:\\Users\\Administrator\\Desktop\\lusc\\8.cox")
rt=read.table("tcga.txt",header=T,sep="\t",check.names=F,row.names=1)
rt[,"futime"]=rt[,"futime"]/365

multiCox=coxph(Surv(futime, fustat) ~ ., data = rt)
#multiCox=step(multiCox,direction = "both")
multiCoxSum=summary(multiCox)

outTab=data.frame()
outTab=cbind(
             coef=multiCoxSum$coefficients[,"coef"],
             HR=multiCoxSum$conf.int[,"exp(coef)"],
             HR.95L=multiCoxSum$conf.int[,"lower .95"],
             HR.95H=multiCoxSum$conf.int[,"upper .95"],
             pvalue=multiCoxSum$coefficients[,"Pr(>|z|)"])
outTab=cbind(id=row.names(outTab),outTab)
write.table(outTab,file="multiCox.xls",sep="\t",row.names=F,quote=F)
```

```
pdf(file="forest.pdf",
	width = 10,			#Width of picture
	height = 5,			#Height of picture
	)
ggforest(multiCox,
		main = "Hazard ratio",
		cpositions = c(0.02,0.22, 0.4),
		fontsize = 0.7,
		refLabel = "reference",
		noDigits = 2)
dev.off()

riskScore=predict(multiCox,type="risk",newdata=rt)
coxGene=rownames(multiCoxSum$coefficients)
coxGene=gsub("`","",coxGene)
outCol=c("futime","fustat",coxGene)
risk=as.vector(ifelse(riskScore>median(riskScore),"high","low"))
write.table(cbind(id=rownames(cbind(rt[,outCol],riskScore,risk)),cbind(rt[,outCol],riskSc
ore,risk)),
	file="risk.txt",
	sep="\t",
	quote=F,
	row.names=F)

setwd("D:\\biowolf\\81immuneLncRNA\\13.survival")
library(survival)
library("survminer")
rt=read.table("risk.txt",header=T,sep="\t")
diff=survdiff(Surv(futime, fustat) ~risk,data = rt)
pValue=1-pchisq(diff$chisq,df=1)
pValue=signif(pValue,4)
pValue=format(pValue, scientific = TRUE)

fit <- survfit(Surv(futime, fustat) ~ risk, data = rt)

pdf(file="survival.pdf",onefile = FALSE,
	width = 6,
	height =5)
ggsurvplot(fit,
		data=rt,
		conf.int=TRUE,
```

```
                  pval=paste0("p=",pValue),
                  pval.size=4,
                  risk.table=TRUE,
                  legend.labs=c("High risk", "Low risk"),
                  legend.title="Risk",
                  xlab="Time(years)",
                  break.time.by = 1,
                  risk.table.title="",
                  palette=c("red", "blue"),
                  risk.table.height=.25)
dev.off()

setwd("C:\\Users\\Administrator\\Desktop\\lusc\\9.distrubi\\15.pheatmap")
rt=read.table("risk.txt",sep="\t",header=T,row.names=1,check.names=F)
rt=rt[order(rt$riskScore),]
rt1=rt[c(3:(ncol(rt)-2))]
rt1=t(rt1)

rt1=-log2(rt1+0.01)
library(pheatmap)
annotation=data.frame(type=rt[,ncol(rt)])
rownames(annotation)=rownames(rt)

pdf(file="heatmap.pdf",width = 10,height = 4)
pheatmap(rt1,
         annotation=annotation,
         cluster_cols = FALSE,
         fontsize_row=11,
         fontsize_col=3,
         color = colorRampPalette(c("green", "black", "red"))(50) )
dev.off()

setwd("C:\\Users\\Administrator\\Desktop\\lusc\\9.distrubi\\16.riskScore")

rt=read.table("risk.txt",header=T,sep="\t",check.names=F,row.names=1)
rt=rt[order(rt$riskScore),]
riskClass=rt[,"risk"]
lowLength=length(riskClass[riskClass=="low"])
highLength=length(riskClass[riskClass=="high"])
line=rt[,"riskScore"]
line[line>10]=10
```

```
pdf(file="riskScore.pdf",width = 12,height = 5)
plot(line,
        type="p",
        pch=20,
        xlab="Patients (increasing risk socre)",
        ylab="Risk score",
        col=c(rep("green",lowLength),
        rep("red",highLength)))
abline(h=median(rt$riskScore),v=lowLength,lty=2)
dev.off()

setwd("C:\\Users\\Administrator\\Desktop\\lusc\\9.distrubi\\17.survStat")

rt=read.table("risk.txt",header=T,sep="\t",check.names=F,row.names=1)
rt=rt[order(rt$riskScore),]
riskClass=rt[,"risk"]
lowLength=length(riskClass[riskClass=="low"])
highLength=length(riskClass[riskClass=="high"])
color=as.vector(rt$fustat)
color[color==1]="red"
color[color==0]="green"
pdf(file="survStat.pdf",width = 12,height = 5)
plot(rt$futime,
        pch=19,
        xlab="Patients (increasing risk socre)",
        ylab="Survival time (years)",
        col=color)
abline(v=lowLength,lty=2)
dev.off()

library(rms)
setwd("C:\\Users\\Administrator\\Desktop\\lusc\\10.nomogram\\NOMOGRAM")
#Set up the working directory
rt=read.table("merger_data.txt",sep="\t",header=T,row.names=1,check.names=F)
#Read input file

#The data package
dd <- datadist(rt)
options(datadist="dd")

#Generating function
```

```
f <- cph(Surv(futime, fustat) ~ ., x=T, y=T, surv=T, data=rt, time.inc=1)
surv <- Survival(f)

#Construction of the nomogram
nom <- nomogram(f, fun=list(function(x) surv(1, x), function(x) surv(3, x), function(x)
surv(5, x)),
        lp=F, funlabel=c("1-year survival", "3-year survival", "5-year survival"),
        maxscale=100,
        fun.at=c(0.9, 0.8, 0.7, 0.6, 0.5, 0.4, 0.3,0.2,0.1,0.05))

#Nomogram visualization
pdf(file="nomogram.pdf",height=6,width=10)
plot(nom)
dev.off()
```

**AUC**

```
setwd("C:\\Users\\Administrator\\Desktop\\lusc\\10.nomogram\\auc")                #Working
directory (to be modified)
library(survival)
library("survminer")
require("survival")
rt=read.table("risk.txt",header=T,sep="\t")
diff=survdiff(Surv(times, status) ~risk,data = rt)
pValue=1-pchisq(diff$chisq,df=1)
#pValue=round(pValue,3)
pValue=signif(pValue,4)
pValue=format(pValue, scientific = TRUE)
Sur <- Surv(rt$times*12,rt$status)
sfit <- survfit(Sur ~ risk,data=rt)
ggsurvplot(sfit,
                conf.int=F, #A confidence interval
                #fun="pct",
                pval=TRUE,
                palette = "jco",
                pval.method = T,
                risk.table =T,
                ncensor.plot = T,
                surv.median.line="hv",
                legend.labs=c("high risk","low risk"))+
                labs(x = "Month")
library("glmnet")
```

```
library("survival")
library("survminer")
library("survivalROC")
library("ggsci")
library("tidyverse")
library("cowplot")
library("pheatmap")
library("ggplot2")
library("Hmisc")
library("plyr")
library("foreach")
library("doParallel")
library("caret")
survivalROC_helper <- function(t) {
  survivalROC(Stime=rt$times*12, status=rt$status, marker = rt$riskscore,
              predict.time =t, method="KM")}
  survivalROC_data <- data_frame(t = c(12,36,60)) %>%
  mutate(survivalROC = map(t, survivalROC_helper),
         auc = map_dbl(survivalROC, magrittr::extract2, "AUC"),
         df_survivalROC = map(survivalROC, function(obj) {
           as_data_frame(obj[c("cut.values","TP","FP")])
         })) %>%
  dplyr::select(-survivalROC) %>%
  unnest() %>%
  arrange(t, FP, TP)
  survivalROC_data1 <- survivalROC_data %>%
  mutate(auc =sprintf("%.3f",auc))%>%
  unite(month, t,auc,sep = " month AUC: ")
  AUC <-factor(survivalROC_data1$month)
  survivalROC_data1 %>%
  ggplot(mapping = aes(x = FP, y = TP)) +
  geom_path(aes(color= AUC))+
  geom_abline(intercept = 0, slope = 1, linetype = "dashed")+
  theme_bw() +
  theme(legend.position = c(0.8,0.2))+
  labs(x = "1-Specificity",y="Sensitivity")+
  scale_color_jco()


setwd("C:\\Users\\Administrator\\Desktop\\survival\\23_Calibration")
#Load installation package
```

```
library(rms)
library(foreign)
library(survival)
seer<-read.table("merger_data.txt",header=T,sep="\t")

#Read the data
seer$risk<-factor(seer$risk,labels=c("≤60",">60"))
seer$race<-factor(seer$race,labels=c("Female","Male"))
seer$Chemotherapy<-factor(seer$Chemotherapy,labels=c("Unmarried","Married"))
seer$pathologic_N<-factor(seer$pathologic_N,labels=c("No","Yes"))
seer$A_sage<-factor(seer$A_sage,labels=c("No","Yes"))

ddist <- datadist(seer)
options(datadist='ddist')
units(seer$survival_time) <- "Year"
fcox <- cph(Surv(survival_time,status) ~ risk + race + Chemotherapy + pathologic_N +
A_sage,surv=T,x=T, y=T,data=seer)
#Calculate one year's standard curve
fcox1 <- cph(Surv(survival_time,status) ~ risk + race + Chemotherapy + Sex +
pathologic_N + A_sage,surv=T,x=T, y=T,time.inc = 1,data=seer)
cal1 <- calibrate(fcox1, cmethod="KM", method="boot", u=1, m=150, B=1000)

plot(cal1)

#Calculate three year's standard curve
fcox3 <- cph(Surv(survival_time,status) ~ risk + race + Chemotherapy + Sex +
pathologic_N + A_sage,surv=T,x=T, y=T,time.inc = 3,data=seer)
cal3 <- calibrate(fcox3, cmethod="KM", method="boot", u=3, m=200, B=100)

plot(cal3)

#Calculate five year's standard curve
fcox5 <- cph(Surv(survival_time,status) ~ risk + race + Chemotherapy + Sex +
pathologic_N + A_sage,surv=T,x=T, y=T,time.inc = 5,data=seer)

cal5 <- calibrate(fcox5, cmethod="KM", method="boot", u=5, m=150, B=100)

plot(cal5)
```
